# *dsdA* Does Not Affect Colonization of the Murine Urinary Tract by *Escherichia coli* CFT073

**DOI:** 10.1371/journal.pone.0138121

**Published:** 2015-09-14

**Authors:** Andrew J. Hryckowian, Gary A. Baisa, Kevin J. Schwartz, Rodney A. Welch

**Affiliations:** Department of Medical Microbiology and Immunology, University of Wisconsin-Madison, Madison, Wisconsin, United States of America; Loyola University Chicago, UNITED STATES

## Abstract

The urinary tract environment provides many conditions that deter colonization by microorganisms. D-serine is thought to be one of these stressors and is present at high concentrations in urine. D-serine interferes with L-serine and pantothenate metabolism and is bacteriostatic to many species. Uropathogenic *Escherichia coli* commonly possess the *dsdCXA* genetic locus, which allows them to use D-serine as a sole carbon, nitrogen, and energy source. It was previously reported that in the model UPEC strain CFT073, a *dsdA* mutant outcompetes wild type in the murine model of urinary tract infection. This “hypercolonization” was used to propose a model whereby UPEC strains sense D-serine in the urinary tract and subsequently up-regulate genes necessary for pathogenesis. Here, we show that inactivation of *dsdA* does not lead to hypercolonization. We suggest that this previously observed effect is due to an unrecognized secondary mutation in *rpoS* and that some D-serine specific effects described in other studies may be affected by the *rpoS* status of the strains used. Inactivation of *dsdA* in the original clinical isolate of CFT073 gives CFT073 Δ*dsdA* a growth defect in human urine and renders it unable to grow on minimal medium containing D-serine as the sole carbon source. However, CFT073 Δ*dsdA* is able to colonize the urinary tracts of CBA/J mice indistinguishably from wild type. These findings indicate that D-serine catabolism, though it may play role(s) during urinary tract infection, does not affect the ability of uropathogenic *E*. *coli* to colonize the murine urinary tract.

## Introduction

Urinary tract infections (UTIs) represent a major public health concern, as they are among the most common human bacterial infections. Forty percent of women and 12% of men will experience a UTI in their lifetimes [1, 2]. Uropathogenic *Escherichia coli* (UPEC) is the most common causative agent of UTIs, representing up to 95% of reported community-acquired cases [3]. UPEC are members of the gut microbiota and gain access to the urinary tract via an ascending route [4]. During this ascent, the bacteria first colonize the periurethral area and traverse the urethra into the bladder, where they cause cystitis. Cystitis can progress to pyelonephritis after the bacteria ascend the ureters into the kidneys of the infected individual.

Although UTIs are common, many conditions in the urinary tract discourage the colonization and growth of microorganisms. Among these stresses are host immune components, physical deterrents, and the chemical composition of urine [5]. D-serine is one of the most abundant amino acids in mammalian urine, where it is present at concentrations from 3–115 μg/ml [6, 7]. D-serine is bacteriostatic, presumably by interfering with pantothenate and L-serine metabolism [8]. High concentrations of D-serine (0.22–14.7 μg/g) are also found in the brain [9], where D-serine acts as a co-agonist for N-methyl-D-aspartate receptors, which are involved in neural transmission and cognitive function [10, 11].

UPEC strains and neonatal meningitis *E*. *coli* (NMEC) strains frequently have the *dsdCXA* locus [12], which allows *E*. *coli* to use D-serine as the sole carbon, nitrogen, and energy source [13]. DsdC is a LysR-type transcriptional regulator [14] that positively affects the transcription of *dsdXA* in the presence of D-serine but in the absence of D-serine, *dsdC* transcription is autorepressed [15–18]. DsdX is a D-serine transporter [19] that belongs to the gluconate permease family of transport proteins [20]. In addition to DsdX, the D-alanine and glycine transporter, CycA, is capable of transporting D-serine [21, 22] and no other D-serine transporters are known in *E*. *coli*. DsdA is a pyridoxal 5’-phosphate-requiring deaminase that is capable of deaminating the D-amino acids D-serine, D-threonine, and D-allothreonine, though its activity is highest towards D-serine and it shows very little activity towards L-enantiomers [23–27].

Based on its commonality in UPEC/NMEC and the ability of DsdC, DsdX, and DsdA to sense, transport, and metabolize D-serine, the *dsdCXA* locus was hypothesized to be important for UPEC/NMEC pathogenesis [12]. Counterintuitively, mutational inactivation of *dsdA* gives rise to a hypercolonization phenotype in UPEC strain CFT073, where a *dsdA* mutant is recovered more frequently than wild type in the bladders and kidneys of CBA/J mice at 48 hours post infection of a 1:1 ratio of these strains [28]. This hypercolonization was used to propose a model whereby UPEC senses D-serine as a urinary tract-specific signal to up-regulate genes needed for pathogenesis. Indeed, UPEC use flagella-mediated motility to ascend the urinary tract during infection [29] and the *dsdA* mutant expresses more flagella and is more motile relative to wild type [28]. Additionally, many other virulence determinants including pap pili and hemolysin, are differentially regulated between these strains during UTI [30].

Other effectors of serine metabolism were described to affect urinary tract colonization by CFT073. In addition to a *dsdA* mutant, *dsdC* mutants hypercolonize relative to wild type, suggesting that there are mechanisms of D-serine dependent virulence gene expression that are independent of DsdC [31]. SdaA and SdaB are L-serine deaminases made by *E*. *coli* [32]. A *dsdA sdaAB* triple mutant is outcompeted by wild type in the bladders and kidneys during murine model UTI, suggesting that DL-serine catabolism is important for colonization of the urinary tract [31]. D-serine transport affects colonization by CFT073 as well: a *cycA dsdXA* triple mutant, which is unable to transport or degrade D-serine, shows a competitive defect in the kidneys of CBA/J mice. This suggests that the expression of traits needed for the colonization of kidneys is aided by the uptake of D-serine [31].

D-serine metabolism is also important for UTI by *Staphylococcus saprophyticus* [33]. Unlike UPEC isolates, *S*. *saprophyticus* does not have the *dsdCXA* locus. It has *dsdA*, which is apparently under the control of its own promoter. *S*. *saprophyticus* Δ*dsdA* is outcompeted by wild type in the bladders and kidneys of C3H/HeN mice. Furthermore, in *S*. *saprophyticus*, D-serine can be used as the sole carbon source during growth, D-serine metabolism provides a growth advantage in vitro, and the expression of the lipase *ssp* [34], is up-regulated in the presence of D-serine. Although UPEC and *S*. *saprophyticus* both have *dsdA*, it appears that the accumulation of D-serine in each of these uropathogens during infection has largely different effects. Aside from UPEC and *S*. *saprophyticus*, D-serine metabolism has not been characterized in any other uropathogens.

Transcription in *E*. *coli* is catalyzed by RNA polymerase holoenzyme, which is comprised of core polymerase and a dissociable sigma factor. *E*. *coli* has one housekeeping sigma factor (σ^70^) and six alternative sigma factors [35]. Core polymerase is unable to initiate transcription alone and requires an associated sigma factor to define promoter specificity and initiate transcription. RpoS (σ^S^), the best studied of the alternative sigma factors, affects the expression of ~10% of genes in *E*. *coli* K-12, either directly or indirectly [36]. Mutations in *rpoS* have been demonstrated in accordance with the transfer of *E*. *coli* strains between laboratories, which is likely due to the growth advantage in stationary phase (GASP) phenotype afforded by loss of function mutations in *rpoS* [37–39].

Here, we show that the strains used in the characterization of the *dsdA* hypercolonization phenotype in CFT073 are not isogenic for *rpoS*. In the original clinical isolate of CFT073, where *rpoS* is intact, mutational inactivation of *dsdA* has no effect on the ability of CFT073 to colonize the murine urinary tract. We propose additional experiments that will allow for a better understanding of the role that D-serine plays in UPEC pathogenesis.

## Results

### The sequenced CFT073 strain has a frameshift mutation in *rpoS*

We previously reported the genome sequence of UPEC strain CFT073 [40], which was isolated from the blood of a pyelonephritis patient in the late 1980s [41]. Upon examination of the CFT073 genome sequence, we observed that this isolate of CFT073 has no annotated *rpoS* gene. However, two annotated ORFs from this strain (c3307 and c3306) each share 99% nucleotide sequence identity with *rpoS* from K-12. Upon further examination of these ORFs, we noticed that there is a 5 base-pair duplication (TAGAG) at the 3’ end of c3307, which leads to a frameshift (amber mutation) in the *rpoS* gene of this strain. Herein, we refer to the sequenced CFT073 isolate as CFT073 *rpoS*_am_ to reflect this amber mutation.

**Fig 1:**
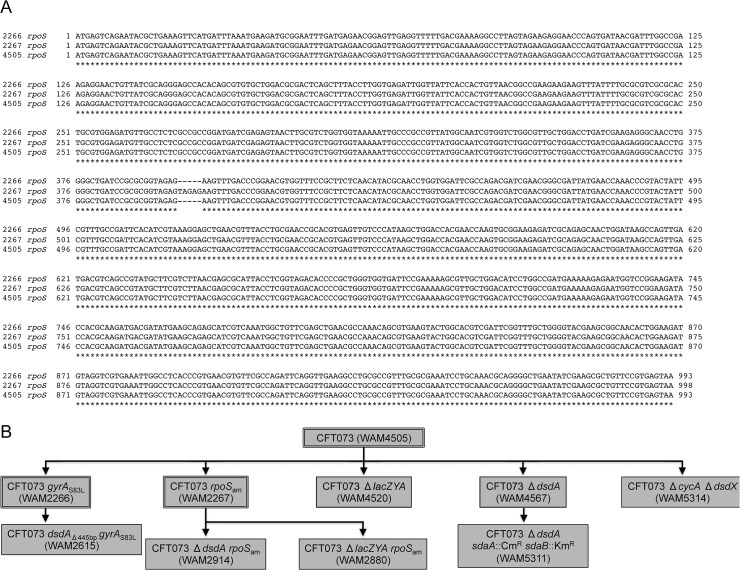
A frameshift mutation is present in *rpoS* from CFT073 *rpoS*_am_ and strain derivatives thereof. (A) The *rpoS* allele from CFT073 (WAM4505), CFT073 *gyrA*_S83L_ (WAM2266), and CFT073 *rpoS*_am_ (WAM2267) was sequenced via Sanger sequencing using the “*rpoS* sequencing F” and “*rpoS* sequencing R” primers listed in Table 1 and an alignment of the *rpoS* coding regions from these strains was performed using the ClustalW function in MacVector 9.0.2. (B) Dendrogram showing relevant strains used in this study (Also listed in Table 1) and their applicable characteristics. Strains mentioned in Panel A are outlined with a double black box.

We determined the nucleotide sequence of the *rpoS* allele from CFT073 *rpoS*_am_ and from a spontaneous nalidixic acid resistant mutant of CFT073 (CFT073 *gyrA*_S83L_) [42] via Sanger sequencing. Because CFT073 *gyrA*_S83L_ colonizes mice indistinguishably from its nal^S^ parent [42], CFT073 *gyrA*_S83L_ strain was previously used as wild type to facilitate enumeration of wild type/mutant ratios during competitive murine model UTI [28, 43]. We found that CFT073 *rpoS*_am_, but not CFT073 *gyrA*_S83L_, has the defective *rpoS*_am_ allele (Fig 1A).

Because *rpoS* mutations arise after prolonged culture in stationary phase and passage between laboratories, we wanted to determine if the original patient isolate of CFT073 has a functional *rpoS* allele. We obtained this isolate from Harry L. T. Mobley (University of Michigan) and showed that *rpoS* is intact in this strain (Fig 1A). We have since deposited the original *rpoS* ^+^ patient isolate of CFT073 in the American Type Culture Collection as ATCC# BAA-2503. This strain is intended to replace ATCC# 700928 (CFT073 *rpoS*_am_) as wild type in all future research. Fig 1B shows a dendrogram of strains used in this study, created based on differences relative to the original patient isolate.

### Hypercolonization is independent of *dsdA*

**Fig 2:**
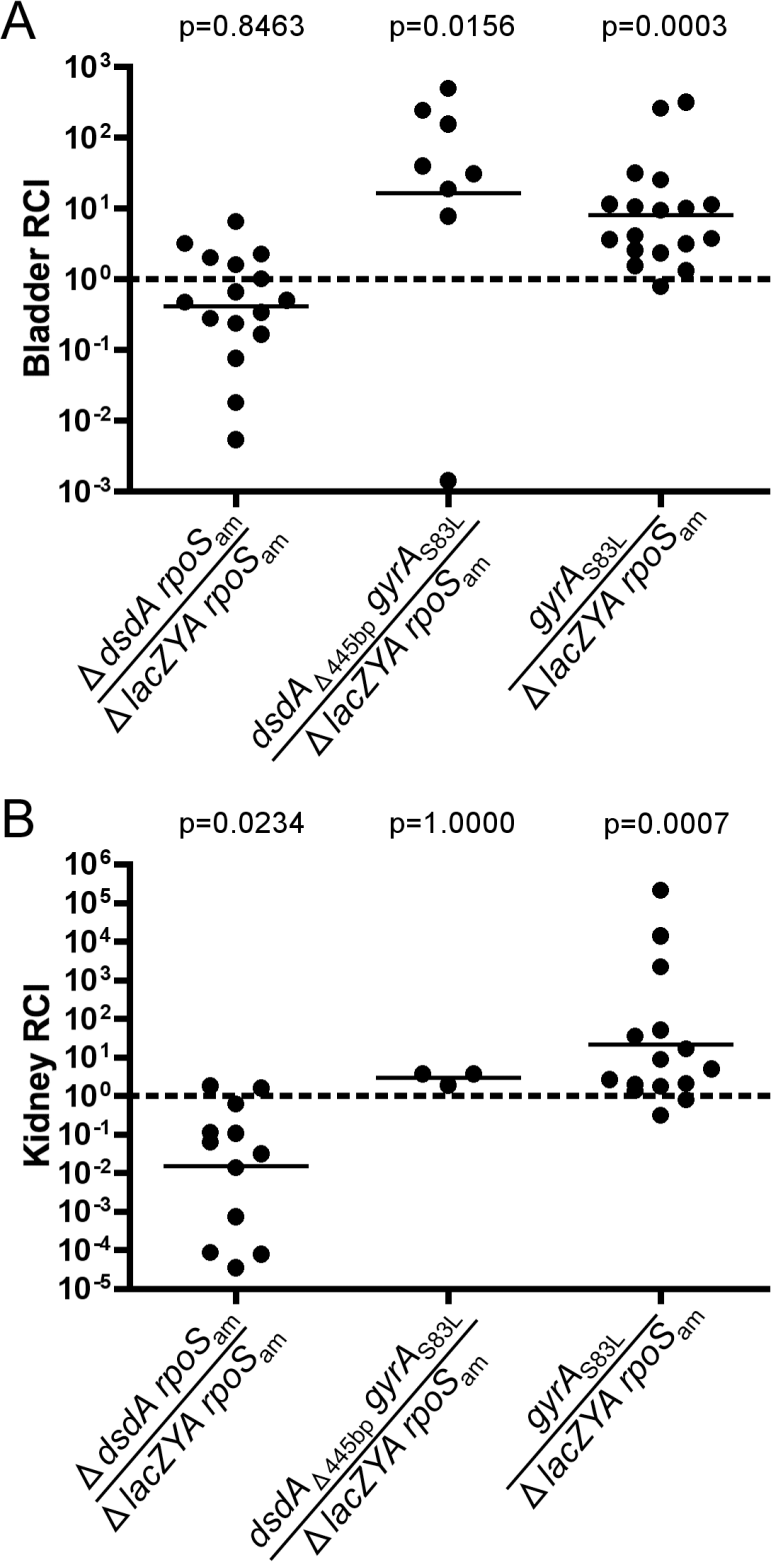
Inactivation of *dsdA* does not engender a competitive advantage in CBA/J mice during experimental UTI. CFT073 Δ*lacZYA rpoS*_am_ and CFT073 Δ*dsdA rpoS*_am_ or CFT073 *dsdA*_Δ445bp_ *gyrA*_S83L_ or CFT073 *gyrA*_S83L_ were co-inoculated at a 1:1 ratio into CBA/J mice (n = 16, 8, and 18, respectively). Mice were sacrificed at 48hpi. Bacteria from (A) bladder and (B) kidney homogenates were enumerated on MacConkey’s medium plus lactose. Several mice from the co-infections had no detectable bacteria in their kidneys (4 of 16, 5 of 8, and 3 of 18, respectively). Lines are drawn at the geometric mean relative competitive index (RCI). Statistical Significance was assessed by a Wilcoxon signed-rank test relative to a hypothetical RCI of 1.

Because the *dsdA* mutant and wild type strains used to describe the *dsdA* hypercolonization phenotype are not isogenic for *rpoS*, we investigated whether *rpoS* affects the *dsdA* hypercolonization phenotype. To this end, we subjected CBA/J mice to competitive UTI using strains containing *dsdA* and *rpoS*_am_ mutations. We then analyzed bacterial burdens in their bladders and kidneys at 48 hpi (Fig 2). First, we showed that CFT073 Δ*dsdA rpoS*_am_ competed equally well with an *rpoS*_am_ strain in the bladders of these animals. As was observed previously [28], CFT073 *dsdA*_Δ445bp_ *gyrA*_S83L_ outcompeted an *rpoS*_am_ strain in the bladders (Fig 2A). However, the *dsdA* ^+^ parental strain (CFT073 *gyrA*_S83L_) also outcompeted an *rpoS*_am_ strain in the bladders and kidneys (Fig 2). CFT073 *dsdA*_Δ445bp_ *gyrA*_S83L_ did not outcompete an *rpoS*_am_ strain in the kidneys. However, kidney RCIs from the two competitive infection experiments using CFT073 Δ*dsdA rpoS*_am_ and CFT073 *gyrA*_S83L_ demonstrate that the lack of *dsdA* does not provide a fitness advantage in this niche (Fig 2B).

**Fig 3:**
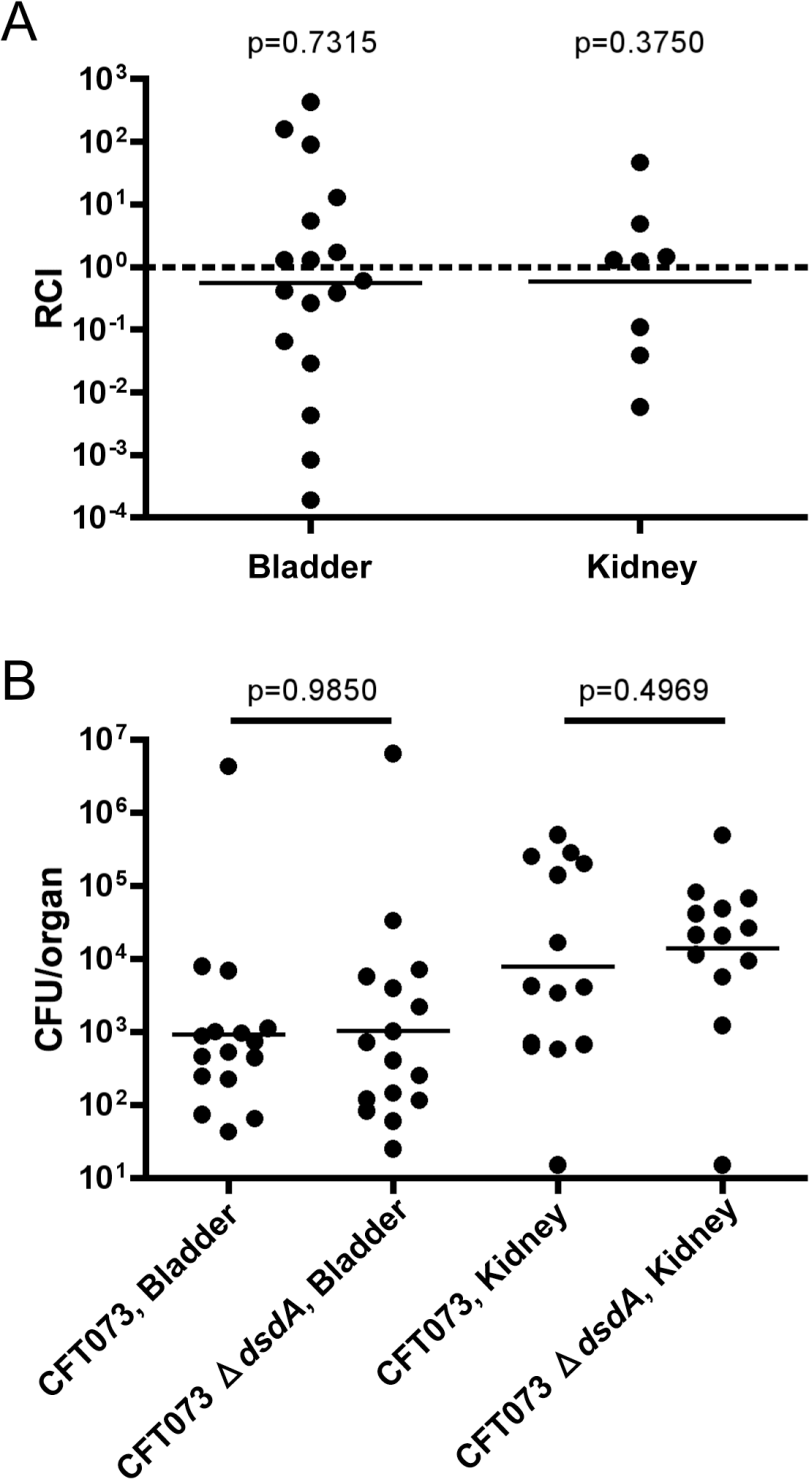
CFT073 Δ*dsdA* colonizes the murine urinary tract indistinguishably from wild type. (A) CFT073 Δ*dsdA* and CFT073 Δ*lacZYA* were co-inoculated at a 1:1 ratio into CBA/J mice (n = 17). Bacteria from bladder and kidney homogenates were enumerated on MacConkey’s medium plus lactose. Lines are drawn at the geometric mean relative competitive index (RCI). Nine mice had no detectable bacteria in their kidneys. Statistical Significance was assessed by a Wilcoxon signed-rank test relative to a hypothetical RCI of 1. (B) Manipulations were carried out as described for panel A except that single strains were used (n = 16 for each). Two mice infected with CFT073 had no detectable bacteria in their kidneys and three mice infected with CFT073 Δ*dsdA* had no detectable bacteria in their kidneys. Lines are drawn at the geometric mean CFU/organ. Statistical significance was assessed by the Mann-Whitney *U* test.

We deleted *dsdA* in the original patient isolate of CFT073 and subjected it to analysis via murine model UTI as above (Fig 3A). CFT073 Δ*dsdA* does not outcompete wild type in the bladders or kidneys of CBA/J mice at 48hpi. Additionally, there is no difference in bladder or kidney burdens in mice subjected to single-strain challenge with CFT073 or CFT073 Δ*dsdA* (Fig 3B).

### *dsdA* does not affect motility in CFT073

We analyzed the motility of the *dsdA* mutants and their parental strains by measuring diameters of migration on Adler’s motility medium (S1 Fig), as described in Materials and Methods. Zones of migration from five independent spots of each *dsdA* ^+^ strain (CFT073, CFT073 *gyrA*_S83L_, and CFT073 *rpoS*_am_) and each *dsdA* ^-^ strain (CFT073 Δ*dsdA*, CFT073 *dsdA*_Δ445bp_ *gyrA*_S83L_, and CFT073 Δ*dsdA rpoS*_am_) were measured. It was determined that *dsdA* does not affect motility under these conditions. Under the conditions tested, strains containing the *rpoS*_am_ allele are significantly less motile than strains that are wild type for *rpoS* (S1 Fig).

### *dsdA* affects the growth of CFT073 in human urine

**Fig 4:**
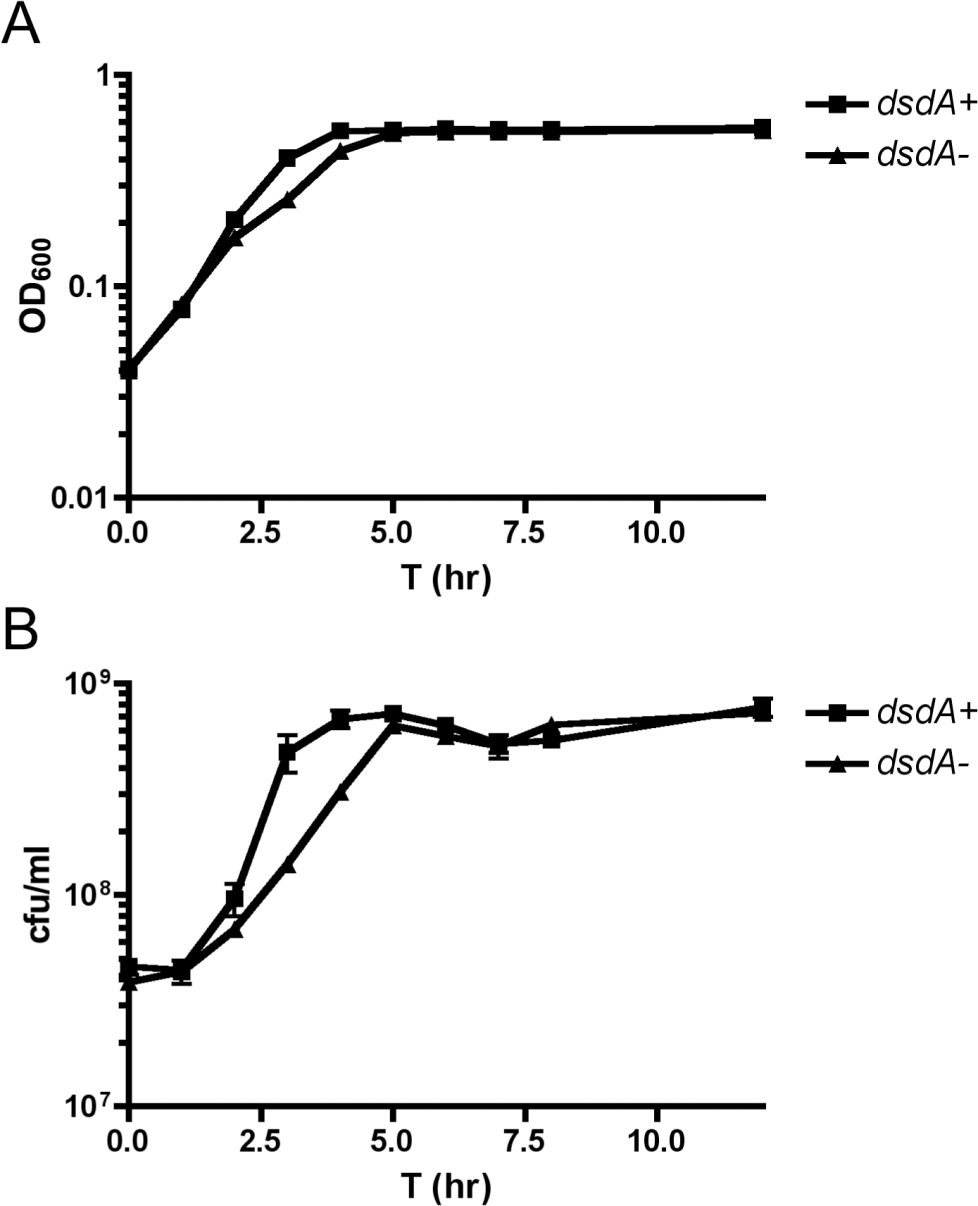
*dsdA* ^-^ strains have a growth defect in human urine. Bacteria were grown overnight in pooled, filter sterilized human urine. Bacteria were washed 2x in phosphate buffered saline and the OD_600_ of each cell suspension was normalized to OD_600_ = 1.5. Bacteria were then inoculated into fresh, pre-warmed urine to OD_600_ = 0.03. Bacteria were allowed to grow for 12 hours. OD_600_ (Panel A) and viable counts (Panel B) were measured at the time points indicated above. *dsdA* ^+^ strains used in this analysis are CFT073, CFT073 *gyrA*_S83L_, and CFT073 *rpoS*_am_, and *dsdA* ^-^ strains are CFT073 Δ*dsdA*, CFT073 *dsdA*_Δ445bp_ *gyrA*_S83L_, and CFT073 Δ*dsdA rpoS*_am_. Data points represent mean OD_600_ and mean viable counts for each strain type where applicable and error bars are drawn to represent standard error of the mean (±SEM).

It was previously shown that a *dsdA* mutant has a prolonged lag phase relative to an *rpoS* mutant when grown in human urine. This lag phase was associated with a significant reduction in viable bacterial counts. However, an increased growth rate was observed at 10–12 hours post inoculation, with equal viable CFUs between strains at 11 hours post inoculation [28]. To reproduce the growth defect that *dsdA* ^-^ strains have when grown in urine, we grew *dsdA* ^+^ strains (CFT073, CFT073 *gyrA*_S83L_, and CFT073 *rpoS*_am_) and *dsdA* ^-^ strains (CFT073 Δ*dsdA*, CFT073 *dsdA*_Δ445bp_ *gyrA*_S83L_, and CFT073 Δ*dsdA rpoS*_am_) in filter sterilized human urine. All *dsdA* ^-^ strains had comparably reduced steady-state growth rate but achieved culture density indistinguishable from that of *dsdA* ^+^ strains after 5 hours (Fig 4). Additionally, the *dsdA* ^-^ strains were unable to grow on MOPS minimal medium containing D-serine as the sole carbon source (S2 Fig).

### Additional effects of D-serine transport and DL-serine metabolism on colonization during murine model UTI

Previously, Anfora et al. reported that D-serine transport and DL-serine catabolism were important for colonization of the murine urinary tract [31]. First, in the *rpoS*_am_ background, a strain unable to transport or degrade D-serine showed a competitive defect in the kidneys of CBA/J mice relative to the parent strain. It was hypothesized that the expression of traits needed for the colonization of kidneys is aided by the uptake of D-serine. To further address this hypothesis in the wild type CFT073 background, we co-challenged CBA/J mice with CFT073 Δ*cycA* Δ*dsdX* and CFT073 Δ*lacZYA*. At 48 hpi, CFT073 Δ*cycA* Δ*dsdX* is outcompeted by wild type 1.67-fold but there is no difference in colonization of the kidneys in these animals (S3 Fig). Anfora et al. also reported in that study that a strain unable to catabolize DL-serine is outcompeted by an *rpoS* mutant, suggesting that DL-serine catabolism is needed by UPEC during UTI. We therefore addressed the importance of DL-serine catabolism in the wild type CFT073 background where, as is shown in S3 Fig, a strain defective for DL-serine catabolism shows a competitive defect in the bladders and kidneys of these animals during experimental UTI.

## Discussion

We discovered that the “wild type” CFT073 strains previously used in our laboratory were either *rpoS* ^+^ (CFT073 *gyrA*_S83L_ and derivatives) or had a frameshift mutation in *rpoS* (CFT073 *rpoS*_am_ and derivatives) and that the original patient isolate of CFT073 has a functional *rpoS* allele (Fig 1). Subsequently, using strains isogenic for *rpoS*, we were unable to reproduce the *dsdA* hypercolonization phenotype that was observed in our laboratory by Roesch et al. [28]. Using murine model UTI, we determined that *dsdA* neither positively nor negatively affects urinary tract colonization by CFT073 (Figs 2 and 3). We also show that *dsdA* does not affect motility (S1 Fig) and that *dsdA* ^-^ strains have a growth defect in urine relative to *dsdA* ^+^ strains (Fig 4). The urine-specific growth defect that we observe here is not as pronounced as what was described previously. We suspect that this is due to the inconsistent composition of urine between collections. Indeed, there is inter- and intra-personal variation in the chemical composition of urine with respect to D-serine concentrations (3–115 μg/ml) and for other urine components [6, 7, 44]. We suggest here that the findings from the previous study by Roesch et al. are due to defects by a strain with the non-functional *rpoS*_am_ allele. Additionally, Anfora et al. showed that CFT073 mutants unable to sense or metabolize D-serine have a competitive advantage over a CFT073 *rpoS*_am_ strain at 48hpi in murine model UTI [31]. We expect that the hypercolonization by these strains is also due to *rpoS* and not the lack of *dsdC* or *dsdA*.

Our findings affect the interpretation of work by Haugen et al., where genes needed for hypercolonization by a *dsdA* mutant over an *rpoS* mutant were identified [30]. Because the strains used in that study were not isogenic for *rpoS* ([30] and Fig 1), it is unclear which genes are differentially regulated in a σ^S^-dependent, DsdA-dependent, or σ^S^/DsdA co-dependent fashion. Interestingly, our data suggest that a σ^S^-dependent mechanism of mitigating the bacteriostatic effects of D-serine is utilized by UPEC during pyelonephritis. Specifically, in Figs 2B and 3A, we demonstrate that a *dsdA* is important for kidney infection, exclusively in an *rpoS* mutant background. As such, a comparison of the transcriptomes of CFT073, CFT073 Δ*rpoS*, and CFT073 Δ*dsdA* during experimental UTI will allow for a better understanding of these effects.

The roles played by DL-serine metabolism and D-serine transport during UTI were also investigated by Anfora et al. [31] using strains not isogenic for *rpoS*. There, it was found that intracellular accumulation of DL-serine, but not the accumulation D-serine or L-serine alone, negatively affects UPEC fitness during UTI. Using strains that are isogenic for *rpoS*, we demonstrate that a *dsdA sdaAB* triple mutant is outcompeted by wild type in the bladders and kidneys of CBA/J mice (S3 Fig), further supporting the findings that DL-serine catabolism, but not D-serine catabolism alone is needed during UTI [31]. Also, using a pair of strains both containing the non-functional *rpoS*_am_ allele, it was shown that a strain unable to transport D-serine [19] competed equally well with its parent strain in the bladders but was at a competitive defect in the kidneys of CBA/J mice at 48hpi. These results suggested that the expression of traits needed for kidney colonization was aided by the uptake of D-serine. However, we demonstrate that CFT073 strains containing the functional *rpoS* allele, the ability to transport D-serine is dispensable for colonization of bladders and kidneys during competitive murine UTI (S3 Fig). This provides evidence that the previously observed defect in kidney colonization was influenced by the *rpoS*_am_ allele and it supports our hypothesis that a σ^S^-dependent mechanism of mitigating the effects of D-serine is utilized by UPEC during pyelonephritis.

This study directly addresses phenotypes reported by Roesch et al. in 2003 and by Anfora et al. in 2007 [28, 31] using the original clinical isolate of CFT073 and isogenic mutants. We extend our findings to inform further commentary on the studies by Anfora et al. and Haugen et al. [30, 31]. We provide evidence that many of the conclusions drawn in these studies are more accurately described in the context of the *rpoS* status of the strains used. As functional σ^S^ is needed during UTI [45], we suggest that the previously described hypercolonization phenotype of a *dsdA* ^*-*^ strain is more accurately described as a colonization defect by an *rpoS* ^-^ strain. The results of this work led us to study the roles and regulation of *rpoS*, which we continue to investigate in UPEC and other *E*. *coli* pathotypes [46].

## Materials and Methods

### Ethics Statement

This study was done in strict agreement with the recommendations found in the Guide for the Care and Use of Laboratory Animals of the National Institutes of Health. The murine model UTI protocol was approved by the UW-Madison Animal Care and Use Committee (Permit Number: M00450-0-07-08). All efforts were made to minimize suffering. Human urine samples were collected from volunteers who gave written consent. Permission for collection of human urine was obtained from the University of Wisconsin Health Sciences Institutional Review Board (IRB). All samples were obtained and used at the University of Wisconsin-Madison.

### Strains, plasmids, and oligonucleotides

Strains, plasmids, and oligonucleotides used in this study are listed in Table 1. PCR amplification of DNA was done using GoTaq (Promega) according to the manufacturer’s specifications. CFT073 Δ*dsdA* was constructed using the Lambda Red mutagnesis protocol [47], which was modified to incorporate a generalized transduction step using ΦEB49 [48] prior to pCP20-mediated antibiotic resistance cassette removal, as described previously [45]. CFT073 Δ*cycA* Δ*dsdX* was constructed by ΦEB49-mediated generalized transduction of *cycA*::Km^R^ from CFT073 *cycA*::Km^R^ *rpoS*_am_ and *dsdX*::Cm^R^ from CFT073 *dsdX*::Cm^R^ *rpoS*_am_ into CFT073, followed by pCP20-mediated antibiotic cassette removal. CFT073 Δ*dsdA sdaA*::Cm^R^ *sdaB*::Km^R^ was constructed by ΦEB49-mediated generalized transduction of *sdaA*::Cm^R^ and *sdaB*::Km^R^ from CFT073 *sdaA*::Cm^R^ *sdaB*::Km^R^ *rpoS*_am_ into CFT073 Δ*dsdA*.

**Table 1:** Strains, plasmids, and oligonucleotides used in this study.

Strain, plasmid, or oligo	Relevant characteristics or sequence	Source
WAM2266	CFT073 *gyrA*_S83L_, nal^R^	H. Mobley
WAM2267	CFT073 *rpoS*_am_	H. Mobley
WAM2615	WAM2266 derivative, *dsdA*_Δ445bp_ *gyrA*_S83L_, nal^R^	[28]
WAM2880	WAM2267 derivative, CFT074 Δ*lacZYA rpoS*_am_	[50]
WAM2914	WAM2267 derivative, CFT073 Δ*dsdA rpoS*_am_	P. Roesch
WAM2966	WAM2267 derivative, CFT073 *dsdX*::Cm^R^ *rpoS*_am_	A. Anfora
WAM3707	WAM2267 derivative, CFT073 *sdaA*::Cm^R^ *sdaB*::Km^R^ *rpoS*_am_	A. Anfora
WAM4248	WAM2267 derivative, CFT073 *cycA*::Km^R^ *rpoS*_am_	G. Baisa
WAM4505	CFT073, original patient isolate	H. Mobley
WAM4520	WAM4505 derivative, CFT073 Δ*lacZYA*	[45]
WAM4567	WAM4505 derivative, CFT073 Δ*dsdA*	This study
WAM5311	WAM4505 derivative, CFT073 Δ*dsdA sdaA*::Cm^R^ *sdaB*::Km^R^	This study
WAM5314	WAM4505 derivative, CFT073 Δ*cycA* Δ*dsdX*	This study
ΦEB49	Generalized transducing phage	[48]
pKD46	Expresses λ RED recombination functions, Ap^R^	[47]
pKD3	Template for λ RED-mediated replacement, Cm^R^	[47]
pKD4	Template for λ RED-mediated replacement, Km^R^	[47]
pCP20	Encodes FLP recombinase, Ap^R^	[47]
*cycA* F Check	5’-GAC TAT CCC GCA GGA ACT GG-3’	Our laboratory
*cycA* R Check	5’-CGG CAT TAA TGA ACT GAT TGA TGA C-3’	Our laboratory
*dsdA* λ RED F	5’-CCT GCT GTC ATT TAT CAT CTA AGC GCA AAG AGACGT ACT TGT GTA GGC TGG AGC TGC TTC G-3’	This study
*dsdA* λ RED R	5’-CAC CCA GGG AAA GGA TGG CGA TGC TGC GTT GAA ACG TTA CAT ATG AAT ATC CTC CTT AG-3’	This study
*dsdA* F Check	5’-GGT TCC GGT GCG ATT GGC TGC-3’	This study
*dsdA* R Check	5’-GGA TGG CGA TGC TGC GTT G-3’	This study
*dsdX* F Check	5’-GAA TTG GTC TGA CAC TTC AAC GCT GC-3’	Our laboratory
*dsdX* R Check	5’-GCA ACC AGT TCT GAT TCA ATA ATC CCC-3’	Our laboratory
*rpoS* sequencing F	5’-CTG AGT GCC TAC GCC CAT AAC GAC-3’	This study
*rpoS* sequencing R	5’-CAA TTA CTG TGC GCT TAA AAT GAT GAT TG-3’	This study
*sdaA* F Check	5’-CGG GAA ACC CTA AAT CAT CGT CAG G-3’	Our laboratory
*sdaA* R Check	5’-GGT TGC GGA AGG GAA TCT ACC A-3’	Our laboratory
*sdaB* F Check	5’-GCG TGG CAA TCA CCA ATA CAG TTG A-3’	Our laboratory
*sdaB* R Check	5’-CGC TAG CCG CGT CTT ATC CG-3’	Our laboratory

### Media and bacterial growth conditions

Strains were grown using the following liquid or solid media: Luria-Bertani (LB) broth or agar, MacConkey’s medium plus lactose, filter sterilized human urine, and 3-(*N*-morpholino)propanesulphonic acid (MOPS) minimal medium [49] supplemented with either glycerol (0.4%) or D-serine (500 μg/ml) as the sole carbon source. Urine was collected and pooled from healthy human volunteers (n = 3) with no recent history of antibiotic use. All strains were grown aerobically at 37°C and supplemented with antibiotics as applicable: kanamycin (Km, 40 μg/ml), carbenicillin (Cb, 250 μg/ml), and chloramphenicol (Cm, 20 μg/ml). In vitro growth analysis in urine was carried out essentially as described previously [28]: bacteria were grown overnight in pooled, filter sterilized human urine at 37°C with aeration, washed twice in phosphate buffered saline (PBS), and added to filter sterilized human urine to an OD_600_ = 0.03. Viable bacterial counts were measured by plating onto LB agar and OD_600_ readings were taken. For growth on minimal media, bacteria were grown overnight on MOPS glycerol plates and single colonies of each strain were swabbed onto both MOPS glycerol and MOPS D-serine minimal media and allowed to grow for 24 hours and 120 hours, respectively.

### Murine Model UTI

Six- to nine-week old female CBA/J mice were used for all experiments described herein. Mice were purchased from Harlan Laboratories (competitive infections) or Jackson Laboratories (single-strain challenge). Mice were either inoculated with a single strain (single infection) or a 1:1 ratio of two strains (competitive infection) as described previously [45] and were sacrificed at 48 hours post infection (hpi). Bladders were homogenized in PBS + 0.0025% Triton X-100 and 10-fold serial dilutions in PBS were plated onto MacConkey’s medium plus lactose. In competitive infections, either CFT073 Δ*lacZYA rpoS*_am_ or CFT073 Δ*lacZYA* was used to facilitate enumeration of wild type/mutant ratios, where applicable. CFT073 Δ*lacZYA* mutants colonize the murine urinary tract indistinguishly from the parental strain [50].

### Motility of *dsdA* mutant strains

Overnight cultures of *dsdA* mutants and their parent strains were plated onto Adler motility medium as described previously [28, 51] with two modifications. First, overnight cultures were washed two times in PBS and OD_600_ was normalized to 1.0 prior to plate inoculation. Second, the diameter of migration from five independent spots was measured after 20 hours of incubation at room temperature. The difference in motility among strains was assessed by one-way analysis of variance (ANOVA) with Tukey’s Multiple Comparison Test to assess statistical significance.

### Statistical analyses

All statistical analyses were carried out using Prism 4.0 (GraphPad, Inc.). Statistical significance was determined by Mann-Whitney *U* test or Wilcoxon signed-rank test for log distributed data where applicable and was determined by unpaired T test or one-way ANOVA for normally distributed data where applicable. P values ≤0.05 represent statistically significant differences between data sets.

## Supporting Information

S1 Fig Mutational inactivation of *dsdA* does not affect motility of CFT073. *dsdA* ^+^ strains (CFT073, CFT073 *gyrA*_S83L_, and CFT073 *rpoS*_am_) and *dsdA* ^-^ strains (CFT073 Δ*dsdA*, CFT073 *dsdA*_Δ445bp_ *gyrA*_S83L_, and CFT073 Δ*dsdA rpoS*_am_) were inoculated onto Adler’s motility medium and zones of migration were measured as described in Materials and Methods. Bars represent mean diameter of migration and error bars represent standard error of the mean (±SEM). The differences in motility between strains were assessed by one-way analysis of variance (ANOVA) with Tukey’s Multiple Comparison Test to assess statistical significance.
(TIF)

S2 Fig *dsdA* ^-^ strains are unable to use D-serine as the sole carbon source. *dsdA* ^+^ strains (CFT073, CFT073 *gyrA*_S83L_, and CFT073 *rpoS*_am_) and *dsdA* ^-^ strains (CFT073 Δ*dsdA*, CFT073 *dsdA*_Δ445bp_ *gyrA*_S83L_, and CFT073 Δ*dsdA rpoS*_am_) were swabbed onto (A) MOPS glycerol minimal medium and (B) MOPS D-serine minimal medium and were incubated aerobically at 37°C for 24 and 120 hours, respectively.
(TIF)

S3 Fig Effects of D-serine transport and DL-serine catabolism on colonization during murine model UTI. CFT073 Δ*lacZYA* was co-inocualted with either CFT073 Δ*dsdA sdaA*::Cm^R^ *sdaB*::Km^R^ or CFT073 Δ*cycA* Δ*dsdX* at a 1:1 ratio into CBA/J mice (n = 10 each). Mice were sacrificed at 48hpi. Bacteria from (A) bladder and (B) kidney homogenates were enumerated on MacConkey’s medium plus lactose. Lines are drawn at the geometric mean relative competitive index (RCI). Statistical significance was assessed by a Wilcoxon signed-rank test relative to a hypothetical RCI of 1. One of the CFT073 Δ*dsdA sdaA*::Cm^R^ *sdaB*::Km^R^/ CFT073 Δ*lacZYA* co-infected animals had no detectable bacteria in her bladder and one of the CFT073 Δ*cycA* Δ*dsdX* / CFT073 Δ*lacZYA* co-infected animals had no detectable bacteria in her kidneys.
(TIF)
