# Supplementary figures and images for: dsdA Does Not Affect Colonization of the Murine Urinary Tract by Escherichia coli CFT073
Source: PLoS One. 2015 Sep 14;10(9):e0138121. doi: 10.1371/journal.pone.0138121 (PMC4569052; doi:10.1371/journal.pone.0138121)

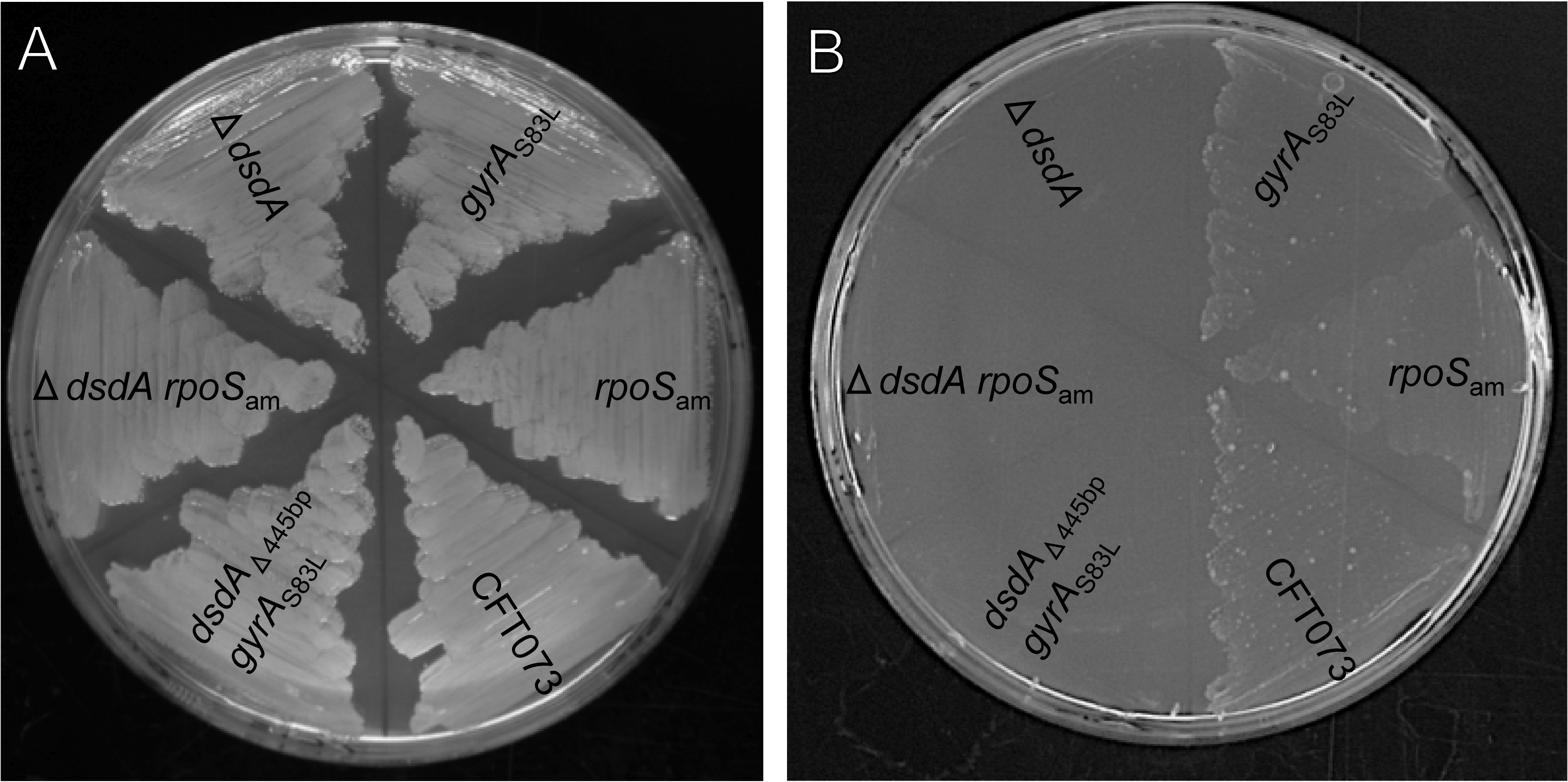

Supplement: S2 Fig — dsdA + strains (CFT073, CFT073 gyrA S83L, and CFT073 rpoS am) and dsdA - strains (CFT073 ΔdsdA, CFT073 dsdA Δ445bp gyrA S83L, and CFT073 ΔdsdA rpoS am) were swabbed onto (A) MOPS glycerol minimal medium and (B) MOPS D-serine minimal medium and were incubated aerobically at 37°C for 24 and 120 hours, respectively. (TIF) [file pone.0138121.s002.tif]

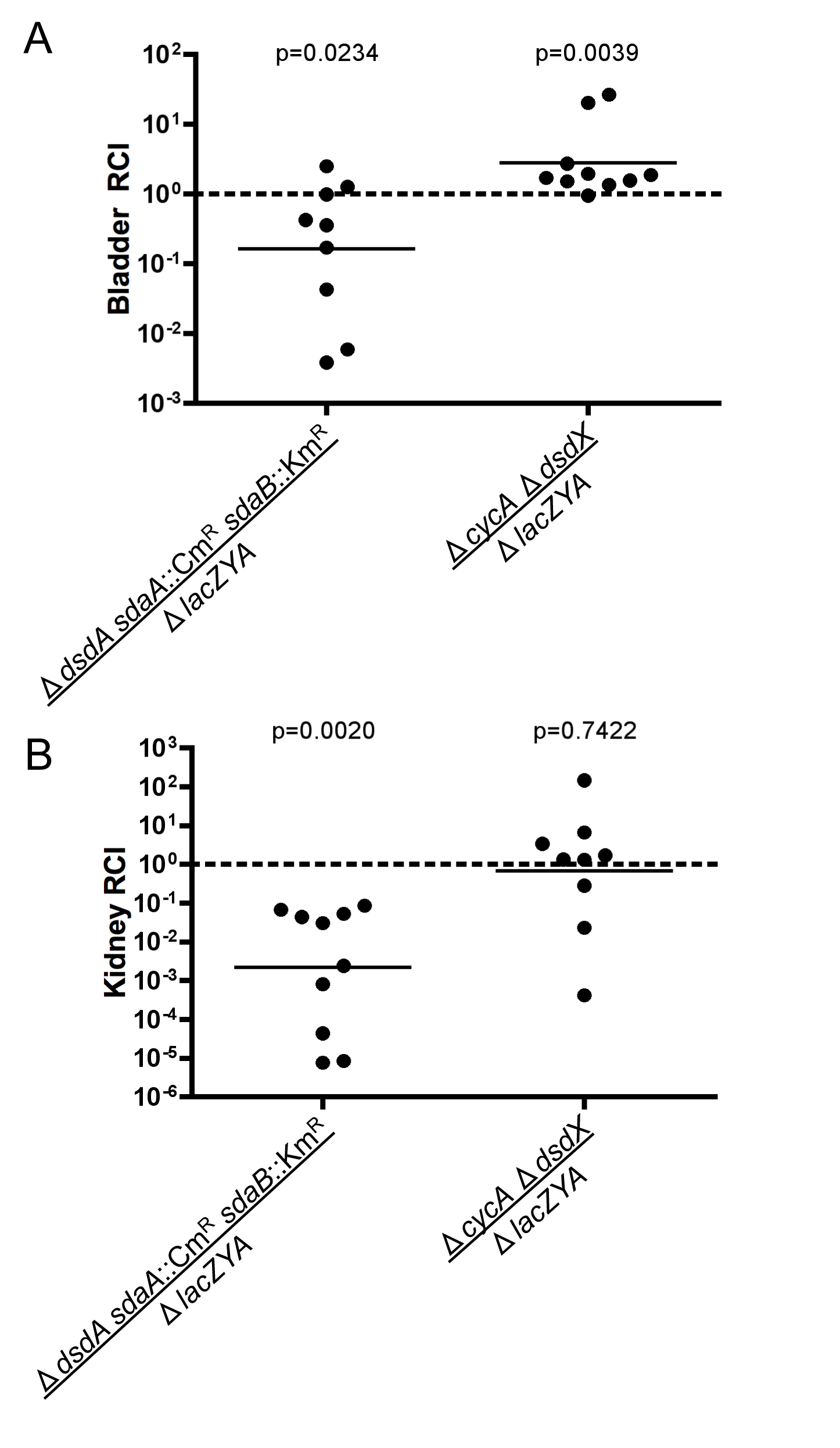

Supplement: S3 Fig — CFT073 ΔlacZYA was co-inocualted with either CFT073 ΔdsdA sdaA::CmR sdaB::KmR or CFT073 ΔcycA ΔdsdX at a 1:1 ratio into CBA/J mice (n = 10 each). Mice were sacrificed at 48hpi. Bacteria from (A) bladder and (B) kidney homogenates were enumerated on MacConkey’s medium plus lactose. Lines are drawn at the geometric mean relative competitive index (RCI). Statistical significance was assessed by a Wilcoxon signed-rank test relative to a hypothetical RCI of 1. One of the CFT073 ΔdsdA sdaA::CmR sdaB::KmR/ CFT073 ΔlacZYA co-infected animals had no detectable bacteria in her bladder and one of the CFT073 ΔcycA ΔdsdX / CFT073 ΔlacZYA co-infected animals had no detectable bacteria in her kidneys. (TIF) [file pone.0138121.s003.tif]
